# A Comparison of Three Airborne Laser Scanner Types for Species Identification of Individual Trees

**DOI:** 10.3390/s22010035

**Published:** 2021-12-22

**Authors:** Jean-François Prieur, Benoît St-Onge, Richard A. Fournier, Murray E. Woods, Parvez Rana, Daniel Kneeshaw

**Affiliations:** 1 Département de Géomatique Appliquée, Centre d’Applications et de Recherches en Télédétection (CARTEL), Université de Sherbrooke, Sherbrooke, QC J1K 2R1, Canada; richard.fournier@usherbrooke.ca; 2 Geophoton Inc., Montreal, QC H3X 2T3, Canada; bso@geophoton.ca; 3 Ministry of Northern Development, Mines, Natural Resources and Forestry (Retired), North Bay, ON P1B 8G3, Canada; woods.murray@gmail.com; 4 Natural Resources Institute Finland (LUKE), P.O. Box 413, FI-90014 Oulu, Finland; parvez.rana@luke.fi; 5 Département des Sciences Biologiques, Université du Québec à Montréal, Montreal, QC H2L 2C4, Canada; kneeshaw.daniel@uqam.ca

**Keywords:** airborne lidar, tree species identification, multispectral lidar, single photon lidar, Random Forest, feature selection, individual tree crown delineation

## Abstract

Species identification is a critical factor for obtaining accurate forest inventories. This paper compares the same method of tree species identification (at the individual crown level) across three different types of airborne laser scanning systems (ALS): two linear lidar systems (monospectral and multispectral) and one single-photon lidar (SPL) system to ascertain whether current individual tree crown (ITC) species classification methods are applicable across all sensors. SPL is a new type of sensor that promises comparable point densities from higher flight altitudes, thereby increasing lidar coverage. Initial results indicate that the methods are indeed applicable across all of the three sensor types with broadly similar overall accuracies (Hardwood/Softwood, 83–90%; 12 species, 46–54%; 4 species, 68–79%), with SPL being slightly lower in all cases. The additional intensity features that are provided by multispectral ALS appear to be more beneficial to overall accuracy than the higher point density of SPL. We also demonstrate the potential contribution of lidar time-series data in improving classification accuracy (Hardwood/Softwood, 91%; 12 species, 58%; 4 species, 84%). Possible causes for lower SPL accuracy are (a) differences in the nature of the intensity features and (b) differences in first and second return distributions between the two linear systems and SPL. We also show that segmentation (and field-identified training crowns deriving from segmentation) that is performed on an initial dataset can be used on subsequent datasets with similar overall accuracy. To our knowledge, this is the first study to compare these three types of ALS systems for species identification at the individual tree level.

## 1. Introduction

Forests are important global resources that affect numerous natural cycles as well as contributing to natural biodiversity, i.e., flora and fauna [1]. Forested lands also constitute the largest terrestrial carbon sink on the planet, with approximate relative contributions of 80% being made by above-ground biomass and 40% being made by below-ground biomass [2].

Forest structural information cannot be fully exploited if species information is missing. Indeed, precise species identification is a crucial variable for forest inventories [3], for the quantification and monitoring of biodiversity [4], and for the study of forest ecosystems and habitats [5]. Accurate tree species identification is the information that is most frequently requested by the forestry industry and by government organisations in the elaboration of forest inventories [6]. However, it is economically unfeasible to sample large numbers of trees in the field. As a consequence, remote sensing is essential not only to supply forest inventories with primary data [7,8,9], but also to address environmental information needs.

High-resolution optical imagery is the most common source of remotely sensed data for species identification. For such images, pixel sizes that are larger than a tree crown may contain foliage from more than one species, leading to ambiguity and frequent identification errors. A small pixel size (e.g., 20–40 cm) implies that a tree crown would necessarily be composed of multiple pixels, leading to a situation where individual pixels will be spectrally representative of neither the tree nor the species. The pixels composing each crown thus include intra-specific spectral variability, which reduces classification accuracy [10]. For this reason, most studies pertaining to tree species identification use object-based classification, which is frequently denoted Individual Tree Crown (ITC) segmentation or delineation [11]. Once the tree crown is delineated, the individual pixels are extracted and summary statistics (e.g., mean spectral signature) and a gamut of features (spectral, spatial, and contextual features, among others) are calculated per crown, which reduces intra-specific spectral variation [12,13]. Optical imaging sensor methods also suffer from major shortcomings when used for species identification at the individual tree level. First, passive optical methods provide information regarding the top of the canopy, especially in dense broadleaf cover, but yield little to no information regarding vertical canopy structure [2]. The second shortcoming of optical methods is related to the anisotropy of reflectance (dependent upon sun-sensor viewing geometry relative to the object) causing different spatial radiometric patterns of the spatial objects (e.g., sun-light vs. shaded crowns) [14,15]. The fact that the bidirectional reflectance distribution function (BRDF) effect is dependent upon the species further complicates the retrieval of information from optical imagery.

Within the broad range of remote sensing technologies that are available to practitioners, airborne laser scanning (ALS) is particularly well adapted to precision forestry, as it provides detailed structural information (given the laser pulse capacity to penetrate closed canopy) [16]. Linear ALS systems are composed of a laser emitter (or multiple emitters in the case of multispectral systems) that produces pulses, which are emitted at a repetition rate of hundreds of kHz. The detector requires a flux of at least 500–1000 photons to register the backscattered laser energy from the target [17]. The detector generates an electronic signal directly and linearly proportional to the backscattered laser energy from the target, hence the name “linear ALS.” The width and amplitude of the returned energy pulse depends upon the target characteristics. Proprietary algorithms transform the multiple peaks of a given waveform into discrete multiple returns. Semi-porous targets such as forest canopies can backscatter multiple peaks corresponding to different components of the canopy (top of crown, leaves, branches, trunk, ground).

The use of ALS data addresses some passive optical sensor limitations that are related to tree species identification. Give that it is an active sensor, lidar signal acquisition is permanently in the hot-spot configuration (the emission angle of the laser pulses is always the same as the viewing angle), which resolves many anisotropy issues [18]. The ALS returns penetrate the canopy to various depths, sometimes reaching the ground. Therefore, the spatial ALS information (i.e., *X*, *Y* and *Z* coordinates) provides species-related structural information concerning the crown, branches and leaves [19]. This canopy penetration and ground resolution capability is the major advantage of linear ALS over other remote sensing methods in the production of enhanced forest inventories.

ALS return intensity values, which are measures of the backscattered laser energy, bring supplemental information about tree species. Intensity values are not only strongly related to the type of foliage [20] and its spectral signature, but the size, orientation, density, and clumping of leaves as well [21,22]. One of the main disadvantages of airborne lidar systems is that there are still many unanswered questions regarding the algorithms that are used to calculate ALS intensity values (they are proprietary to the various instrument manufacturers) and they preclude the comparison of lidar acquisitions that are provided by different sensors and over-flights. Additionally, the linear ALS system used in this study is monospectral, which precludes the use of vegetation indices to improve classification accuracy. In order to address the latter point, multispectral ALS systems is one of the latest major innovations to have developed over the past few years [23]. The three channels with different wavelengths provide additional intensity features and permit the calculation of ratios analogous to NDVI. The intensity comparison issues between different surveys remain with MSL however, as with all lidar systems.

ALS technology is undergoing rapid evolution. One of the most important variables in ALS acquisition specifications is the point density or the average number of returns per m^2^. This density depends upon flight altitude and flight speed for a given pulse repetition frequency. Therefore, there is a direct relationship between the cost ($/km^−2^) of acquisition and the point density. There is also a relationship between ALS point density and classification accuracy for ITC methods. Conversely, methods using the Area-Based Approach (ABA) provide good results at lower point densities and results accuracy that do not necessarily improve proportionally with point density [24]. Even if the importance of these relationships is well known, it remains unclear what effect ALS point density exerts on ITC identification.

As soon as the first commercial linear ALS systems appeared in the mid-1990s, researchers also started to explore the use of photon-counting instruments, i.e., Single-Photon Lidar (SPL), to address some shortcomings of conventional or linear ALS systems such as the high cost to obtain coverage of an area, even when compared to optical imaging sensors. SPL covers larger areas at comparable densities at much higher flight altitude, potentially reducing costs [25]. SPL also provides opportunities for more frequent over-flights. SPL instruments utilise beam energy in a more efficient manner than linear ALS; therefore, the former obtains a higher point density for a given flight altitude than the latter [26]. Alternatively, SPL achieves acceptable point densities while flying at a higher altitude, thereby permitting greater coverage [27]. SPL systems use a laser that is split into a 10 × 10 array of “beamlets” with the return energy being acquired by a 10 × 10 array of single-photon sensitive detectors [28,29]. The intensity value for each SPL return pulse is not well defined and is derived differently from that of linear systems. For example, the data provider for the SPL over-flight that was used in this study uses the pulse width of the returned energy as an analogue of linear lidar intensity. In addition, the return distribution, such as the ratio of first to second returns, is different in the SPL case when compared to linear ALS systems.

The short recovery time of the detector is a crucial element of SPL technology, as it enables multiple close-by photon measurements along the beam’s path for each laser pulse that is emitted. The high sensitivity that is required of the pulse detector to detect single photon returns from the surface also makes it susceptible to background noise; the most important noise source is solar illumination reflecting off said surface [30]. This background noise is proportional to the instrument Field-Of-View (FOV) and to the receiver telescope aperture, both of which are reduced in the type of sensor that is used for this paper. Noise filtering algorithms, such as the Differential Cell Count method, are used to further reduce interference from background solar illumination [31].

Several ABAs that have been developed under linear ALS systems were adapted for use with SPL data to map forest attributes. ABA metrics that were derived under multispectral ALS and SPL systems were comparable [32,33]. The SPL data resulted in slightly better estimates for all canopy structural variables compared to multispectral linear ALS, except for basal area. Since SPL covers 590 km^2^ h^−1^ compared to 50 km^2^ h^−1^ for multispectral linear ALS at equivalent point density, SPL sensors clearly provided a productivity advantage over linear ALS systems for methods using ABA [34]. However, the classification performance of SPL for tree species identification has yet to be ascertained since the SPL point cloud exhibits both a different vertical distribution as well as differences in the ratio between first and second returns compared to linear mode systems.

The main objective of this study is to compare the tree species identification capabilities from three datasets that were acquired respectively with linear monospectral ALS, linear multispectral ALS, and an SPL system. To our knowledge, this is the first study to compare these three types of ALS systems when used for species classification at the individual crown level. In particular, we wish to verify whether the methods that were developed for linear ALS data perform as well with SPL data. Species identification methods were tested at three classification levels: broad species types (hardwood, HW vs. softwood, SW), narrow species groups (e.g., pines, spruces), and specific tree species. A secondary objective was to determine whether an increased number of species identification features that were derived from multispectral lidar or the higher point density of SPL provides greater classification accuracy compared to the standard mono-spectral linear ALS baseline. Finally, additional specific questions were addressed: Are the most relevant features the same for the three sensor types, or do they differ significantly? Does feature selection affect classification accuracy in the same manner for these three datasets?

## 2. Materials

### 2.1. Study Area

**Figure 1:**
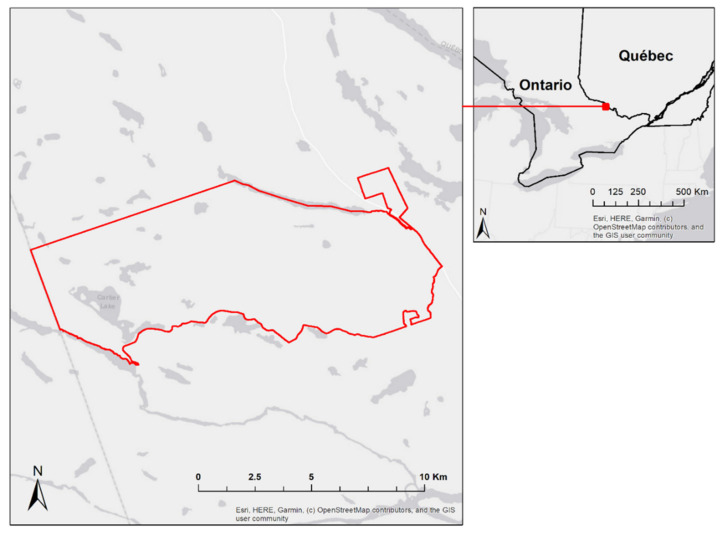
Overview and location of common study area (red outline), Petawawa Research Forest, Ontario, Canada.

The Petawawa Research Forest (PRF) is a 10,000 ha forest that is situated about 200 km NW of the City of Ottawa, ON, Canada. PRF is composed of mixed-mature natural stands as well as plantations and is representative of the Great Lakes-St. Lawrence Forest type [35]. Common species include eastern white pine (*Pinus strobus*), red pine (*Pinus resinosa*), trembling aspen (*Populus tremuloides*), paper or white birch (*Betula papyrifera*), yellow birch (*Betula alleghaniensis*), red maple (*Acer rubrum*), and sugar maple (*Acer saccharum*). Both boreal species and shade-tolerant hardwoods exist throughout the area. The climate of PRF is characterised by a mean annual temperature of 5.6 °C (−11.8 °C in January, 20.3 °C in July), and average annual precipitation of 859 mm, with 682 mm falling as rain and 182 cm as snow [36]. The research forest lies on the southern edge of the Precambrian Shield, with elevations ranging from 140 to greater than 280 m above sea level [37]. Its gentle topography is strongly influenced by glaciation and post-glacial outwashing. Three types of terrain characterise the PRF: extensive sand plains of mostly deltaic origin; imposing hills with shallow sandy soils, as well as bedrock outcrops; and gently rolling hills that are composed of moderately deep, loamy sand that contains numerous boulders. Figure 1 shows the extent of the common study area (line in red) for the three datasets used in this study.

### 2.2. Airborne Laser Scanning Data

Three different datasets were used for this study. First, a monospectral linear ALS (Riegl 680i; 1550 nm) was flown in 2012 (hereafter, designated as **ALS12**), a multispectral linear ALS (Optech Titan; 532, 1064 and 1550 nm) was flown in 2016 (**MSL16**), and a photon-counting lidar (Leica SPL100; 532 nm) was acquired in 2018 (**SPL18**). Information on the respective acquisition parameters and sensors is provided in Table 1. In an ideal situation, the three datasets would have been acquired simultaneously and then compared. Logistical and financial considerations rendered this unpractical. The main difference between the three datasets is the altitude flown during acquisition; 3760 m for SPL compared to 600–750 m for the linear systems. Despite the much higher flying altitude, the point density of SPL remains much higher than that of the other sensors owing to the principle of single photon measurements. The triple-beam configuration of the MSL system provides increased point density (similar to SPL18) when compared to the monospectral ALS system. The use of the 532 nm green wavelength in the SPL system, much like the green channel of the MSL system, hampers pulse penetration in thicker canopies, as witnessed by the much lower point density of the MSL16 green channel compared to the IR channels.

Prior to our use of the information, the ALS12 and SPL18 datasets were processed by their respective vendors to classify the ground/non-ground points using proprietary software. For the multispectral dataset (MSL16), the three channels (C1, C2 and C3) were combined into a single point cloud (C321) for the calculation of the geometric feature set. In contrast, intensity features were calculated per channel, as pooled intensity features would be meaningless. Normalised differences between channels were computed to produce NDVI-like features, as found in [38].

**Figure 2:**
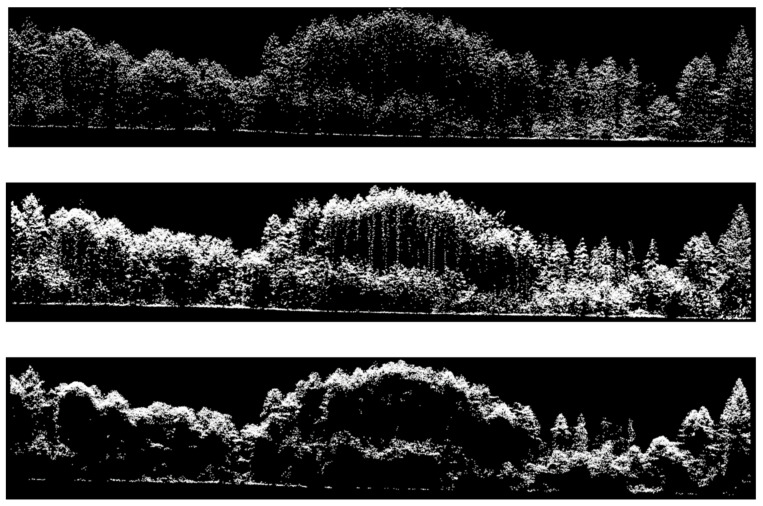
Transect extracted from the three lidar datasets: (**top**) monospectral ALS (ALS12); (**middle**) multispectral ALS (MSL16), with the three channels combined; and (**bottom**) photon-counting lidar (SPL18).

Transects that were taken of the same area, but from the three different point clouds, provide an example of monospectral ALS (ALS12—Figure 2 (top)), multispectral ALS (MSL16—Figure 2 (middle)), and photon-counting lidar (SPL18—Figure 2 (bottom)) datasets. Photon-counting lidar featured a high point density when compared with the other two datasets despite being flown at a higher altitude. Differences in the middle-story and ground hits can also be seen between the three datasets.

## 3. Methods

For the purposes of this study, all processing was performed using in-house software developed in the Python and R languages. This processing ranged from the initial data layers, i.e., the point cloud, digital terrain model (DTM), digital surface model (DSM), and canopy height model (CHM), to feature extraction and balanced Random Forest classification [39]. The species identification methods that are proposed in this article were initially developed for operational deployment with an industrial partner over large (e.g., 200,000 ha) commercial forests. Given this criterion, processing speed was one of the primary drivers guiding method development. This explains, for example, the use of raster-based methods rather than more sophisticated point cloud methods for individual tree crown segmentation, together with the need for feature selection in our Random Forest models. We (and others) [40] have found that parsimonious classification models perform better when they are applied to a large study area, while also making the analysis of selected features easier to implement.

### 3.1. Individual Tree Crown (ITC) Segmentation

As the MSL16 point cloud was not processed to classify ground points, the 2012 point cloud was used to produce the reference DTM. This was generated with Whitebox Tools [41] at a 25 cm-resolution using a Delaunay triangular irregular network fitted to the lidar ground points. The DSM for both the ALS12 and MSL16 dataset was processed using the same algorithm. DTM, DSM, and CHM rasters were provided with the SPL18 dataset. SEGMA (https://en.geophoton.ca/t%C3%A9l%C3%A9chargements (accessed on 17 October 2021)) software v 0.3 [42] was used to delineate the ITCs from the ALS12 CHM. Within SEGMA, the CHM with XY resolution of 0.25 m is first filtered using a Gaussian filter, in which the σ (sigma) value varies proportionally to the local CHM height. Local maxima are then detected on the filtered CHM using an exclusion radius that was proportional to local CHM height; for a local maximum to be detected, a pixel must be higher than all of the pixels that are found within a radius determined by the local height. Regions are grown around these maxima until certain criteria are met, such as reaching a crown height much smaller than the local maximum [42]. At this stage, a certain number of attributes are computed, such as the height (maximum unfiltered CHM height within the crown), crown area, diameter, height-to-area ratio, vertical extent (difference between the highest and lowest unfiltered CHM height in a crown), crown ratio (vertical extent over height), circularity and eccentricity, among others. A delineation score is computed automatically as a weighted mean of these attributes. Crowns having a low delineation score or improbable proportions (e.g., an outlying value of height to area ratio) are resegmented by erosion. The final crowns are polygons that are recorded in a vector layer (shapefile) with their attributes.

After automated delineation, the quality of the ITC was appraised visually by overlaying the delineated crowns onto the CHM or onto ortho-photos to ensure that delineation problems would not compromise subsequent methodological steps. Using visual analysis (which we recognise as being subjective), ITC delineation performance was generally very good, but lower in dense, hardwood-dominated forests. This may have introduced omission and commission errors when identifying tree crowns.

**Figure 3:**
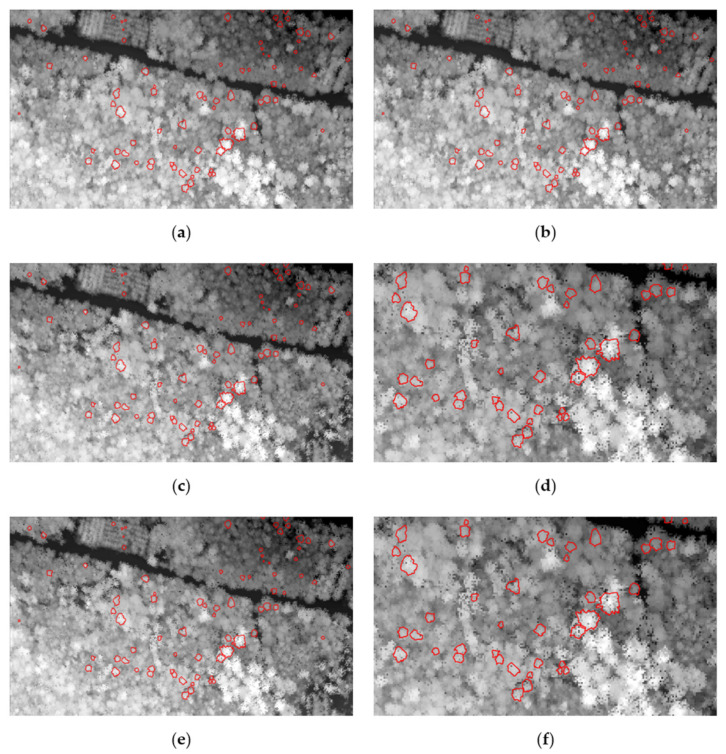
Individual tree crown alignment comparisons for the three datasets: ALS12 (**a**,**b**); MSL16 (**c**,**d**); SPL18 (**e**,**f**). The crown polygons in red are crowns that were delineated on the ALS12 dataset (**a**,**b**), field identified and carried forward for training on the other two datasets. The crowns still align well visually with the respective CHMs.

Crown matching is required to be coherent between datasets. Therefore, crown delineation was performed using SEGMA on the ALS12 dataset. These crown polygons were subsequently used to extract features on all three datasets. Visual inspection of the ALS12 crown outlines (Figure 3a,b) that were overlaid on the MSL16 (Figure 3c,d) and SPL18 (Figure 3e,f) showed that most properly delineated crowns still showed good agreement with the crowns that were visible in the CHM of the two more recent datasets.

### 3.2. Feature Calculation

Geometric and intensity features that were derived from the ALS points of each delineated crown were used to identify species. The geometric features (based on X, Y and Z lidar data) included tree proportions, vertical crown profile, and porosity to laser pulses, among others. The intensity features were based on measures of central tendency (mean, median) and dispersion (standard deviation) of the laser return intensities. We used a subset of the features that were described by [38]; these are enumerated in Table 2 and Table 3.

In the case of geometric features, it is possible to normalise each laser return elevation to height above ground by subtracting the underlying raster DTM elevations under each ALS return. We avoided this normalisation because this warps the 3D shape of tree crowns in the presence of terrain slope [43]. Instead, we extracted a single DTM value at each crown’s centroid and used this single value to normalise all of the ALS points falling within the corresponding crowns.

The following steps involved extracting the laser returns for each crown and normalising them to the single DTM height. Points below 2 m above ground were discarded. In addition, all geometric features that relate to tree size, e.g., the height at the *i*th percentile, were normalised relative to the tree height, as:

(1) $$F_{n}\left(i\right)=F\left(i\right)/H\left(i\right)$$

where *F_n_* is the normalised feature value based upon the absolute value of *F*, and *H* is the height of the *i*th tree. Calculating *F_n_* ensures that species identification remains independent of the height distribution of trees in the training samples [44]. Adimensional geometric features, such as the ratio of crown area to height, or the slope of the lines connecting the highest return to each of the other returns, were not transformed.

No intensity normalisation was required, since range information was unavailable for all three datasets. Our preliminary tests showed a negligible effect of intensity normalisation using alternative methods to determine range (such as using the above-ground altitude of the aircraft and the scan angle as a proxy for range for the study area) on the classification accuracy of our Random Forest models.

Overall, a total of 34 3D features (all three MSL channels were combined into a single channel for the calculation of these 3D features), and 16 intensity features (65 in the case of MSL where each individual channel was used) were computed for each tree.

### 3.3. Species Classification Model

#### 3.3.1. Training Crown Selection

Reference training crowns were sampled and identified based on ITC delineation (using SEGMA) performed on the ALS12 dataset. An initial set of training crowns (N = 331) was identified in 2014 by trained photo-interpreters with Ontario Ministry of Natural Resources and Forestry (MNRF) with high confidence in conifer identification and good confidence with regard to hardwoods. A second set of training crowns (*n* = 1109) was identified by field crews that were cruising targeted areas to achieve the proper spatial distribution of training crowns in the summer of 2015. For this campaign, field crews cruised the forest with an SX-Blue GNSS receiver that was obtained from Geneq Inc. (Montreal, QC, Canada). The GNSS receiver contained GPS, GLONASS (a Russian satellite-based navigation system), and a Wide Area Augmentation System (WAAS). The WAAS-corrected geo-location was shown on a field tablet displaying the CHM raster and the delineated crown polygons. Matching was sometimes complex because an actual crown may bear little resemblance to the associated polygon; additionally, the field position may drift due to GPS positioning error.

Based upon geo-location-assisted visual association between a tree in the field and its representation on the tablet, the matching crown polygon species label was added to the training crown shapefile on the tablet. The training crowns were curated using recent high-resolution aerial imagery to remove felled or dead crowns. Throughout the sampling campaign, care was applied to gathering trees of different heights, from 5 m to height at maturity for each species. The overall goal was to collect an equal number of sample crowns per species; this proved to be difficult, as abundance varied between species and per stand. Three crowns were removed during a visual quality control step and species with fewer than 40 exemplars were removed. The resulting number of sample crowns per species is presented in Table 4. Due to the complexity and expense related with field training crown selection, it was unfeasible to conduct campaigns for the MSL16 and SPL18 acquisitions; hence, the ALS12 training crowns were used as a reference in this study.

#### 3.3.2. Classification Groupings

We performed four different groupings to compare classification across the three datasets: two tree types (HW/SW); four genera with four species; five functional groups; and a species grouping with twelve species, as seen in Table 5. Differences in species counts reflect the fact that some features in the MSL and SPL datasets cannot be calculated for those crowns; we cannot use crowns with missing values to train our Random Forest models; therefore, they are discarded. This is likely due to the tree having been felled during the time interval between initial acquisition and delineation (2012), or to differences between the features that were calculated depending upon the lidar system being used. For example, the green channel of the MSL system has been shown to attain a lesser degree of penetration than do the other two IR channels [45], resulting in some crowns having no second returns in the MSL acquisition. One type of 3D feature (RM from Table 2) uses second returns, so these features cannot be computed for crowns without second returns. A similar problem exists for SPL systems, since far fewer second returns are recorded by these systems than by linear ALS systems (see Figure 1) [46], resulting in crowns being discarded as well.

#### 3.3.3. Random Forest Training and Feature Selection

The species were identified using a Random Forest (RF) classifier. This classification method offers several advantages compared to other methods. It leads to the best or at least equivalent accuracy when compared to other methods [47]. RF has been found to be well suited for several tree species classification studies [6,22,38,48]. RF has been shown to not rely upon assumptions of normality and homoscedasticity. We applied the Shapiro–Wilk test to our datasets and found that none of the features followed a normal distribution. This lack of normality eliminates widely used parametric statistical tests, such as linear or quadratic discriminant analysis. Finally, RF is able to handle a very large set of predictors and exhibits a low sensitivity to collinearity between features [49] as well as a low propensity to over-fit the model [39]. However, it is sensitive to unbalanced data (such as ours), that include large discrepancies in the number of samples per class. Various sub-sampling strategies can be applied to the training set to balance the classes for model training [50].

The number of geometrical and intensity features that were calculated (as per Section 3.2) resulted in a large feature set. Using the complete feature set (high dimensionality), especially given the paucity of training crowns per species (N = 35 in the worst case, after crowns with missing features are removed), can result in a reduction in prediction power, over-fitting, and a reduction in the generalisability of the models. These problems, particularly the loss of predictive power, exemplify the Hughes effect, or what [51] referred to as “the curse of dimensionality.” Due to the number of features that were calculated, we proceeded with two widely used feature reduction methods: an initial ranking and filtering of all features [52], followed by stepwise selection of the final features. The first criterion that was selected in the initial feature filtering step was the mean decrease in accuracy (MDA) function, found in the Random Forest package [53] of R [54]. Only features with an MDA > 0.1 were retained. Next, features with a correlation > |0.9| with another were removed, retaining the one having greater usefulness (largest MDA value) in the inter-correlated pair.

The Variable Selection Using RF (VSURF) algorithm [55] was then used to perform the final feature selection. VSURF is a wrapper-based algorithm that uses the MDA information contained in the RF model to select features. The desired number of features is ranked based upon MDA scores over 50 permutations; features that include negligible or zero contributions to the classification are removed. The remaining variables are then tested in a variety of RF models with the most accurate model being retained; MDA is only used initially to rank the features. An ascending stepwise function is then used, which removes redundant features based upon their contributions to the out-of-bag (OOB) error. The threshold for rejecting a feature is based upon a function that minimises OOB error. These remaining features were subsequently used to train the RF models for each dataset. As a result, the retained features differed for each dataset, depending upon the usefulness of the features in their respective datasets and their degree of inter-correlation. Retained features were used to construct the final RF model for each species grouping and for each dataset. Due to the heuristic nature of the VSURF algorithm, the resulting feature set is not necessarily the best set of features, but rather a good one to train our models [56]. The resulting models were run 20 times on the training data to calculate the average overall accuracy. A classification was performed and its accuracy was assessed using three feature sets for each dataset: (1) all selected features; (2) the 25 best features; and (3) the 15 best features.

Finally, to understand the respective roles of the 3D and intensity features, we report classification accuracies resulting from using only 3D features, only intensity features, or all features. Furthermore, to explore the advantages of using a combination of systems, and acquisition over multiple years, we combined the features of all systems into a single classification.

## 4. Results

The RF classification accuracies were compared for four different species groupings, three ALS systems (ASL12, MSL16, SPL18), and four broad feature groupings: 3D only; intensity (I) only; all the features of a given ALS system; and all the features of all the ALS systems pooled (Table 6). This comparison was performed following an initial variable selection (based upon MDA, inter-correlation, and VSURF). The best accuracies were achieved for the first level of classification, i.e., the type distinction between hardwood and softwood species, while the lowest accuracies occur at the 12 species level. At the finest classification level, there was a noticeable difference in accuracy between most hardwood (in the grey background of Table 7) and softwood species (in the white background of Table 7) for the best model (all sensors, all features) with eastern larch (*Larix laricina*) being a notable exception to this pattern. This result was not necessarily surprisingly, given that larch is a deciduous softwood.

Multispectral ALS (3D + intensity features) produced the best results in all species groups, and all feature subsets, while SPL ALS displayed a systematically lower accuracy compared to the two other types of sensors. The decrease in performance was almost always greater going from standard ALS to SPL, highlighting the different nature of SPL compared to the two linear ALS systems. However, both linear ALS systems (standard and MSL) generally produced comparable results, with a small advantage being shown by the MSL sensor in most cases.

The relative information contents of the 3D and intensity features varied across systems. Unsurprisingly, the three-wavelength intensity features of MSL provided greater species identification performance than did its 3D features. The reverse was true in the case of the two other systems. In most cases, the contrast between the discrimination power of the 3D and the intensity features was greater in SPL, with the 3D features performing much better than the intensity features. The SPL models displayed two fewer intensity (I_) features than the standard ALS, given that they were more strongly correlated and were removed in the feature selection process. It must be reiterated that SPL intensity is an ill-defined quantity and care must be taken in the interpretation of results that are derived from it. In all cases, the single intensity channel of standard ALS provided greater accuracy than that of the SPL system, while the accuracy that was provided by the 3D features of SPL was similar to that of the other two sensors, or slightly lower.

For each ALS system and each species grouping, the greatest accuracy was attained when the 3D and intensity features were combined. For the simplest classification level (hardwood vs. softwood), the pooled 3D and intensity variables did not feature substantially greater accuracy compared to that of the best subset (intensity-only or 3D-only, depending upon the case). For the most complex level (12 species), the contrast was greater, particularly in the case of standard ALS, where the numbers rose from 38.9% (3D-only) to 50.7% (all).

**Figure 4:**
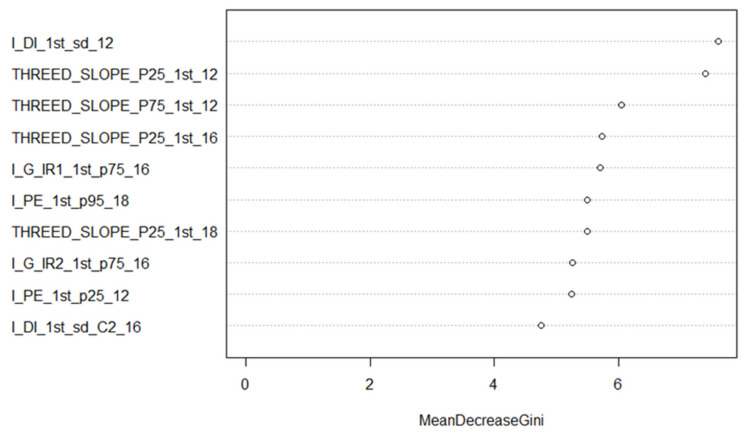
Mean Decrease Gini for the 12 species classification (all sensors, top 10 features). The suffix after each feature (_12, _16, _18) refers to the dataset (ALS12, MSL16, SPL18) from which the feature was calculated.

**Figure 5:**
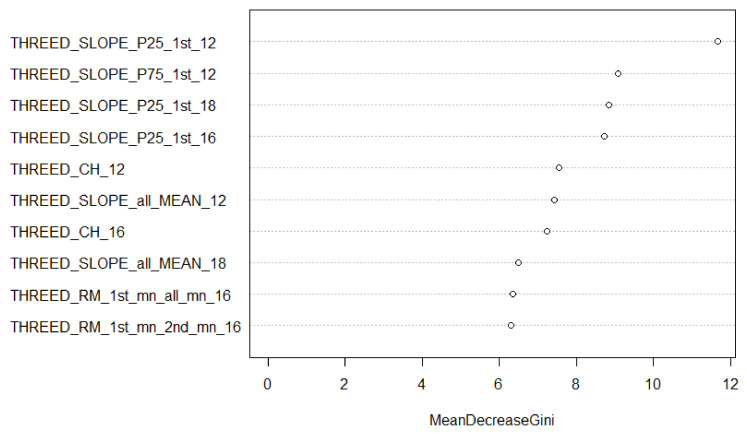
Mean Decrease Gini features for the 12 species classification (all sensors, top 10 3D features only). The suffix after each feature (_12, _16, _18) refers to the dataset (ALS12, MSL16, SPL18) from which the feature was calculated.

**Figure 6:**
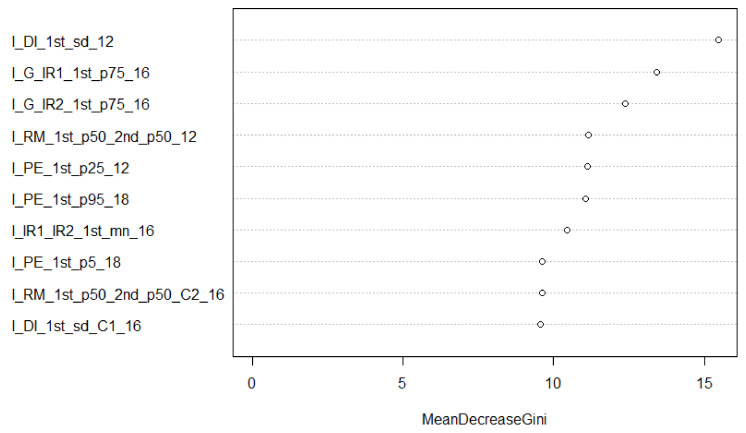
Mean Decrease Gini features for the 12- species classification (all sensors, top 10 I features only). The suffix after each feature (_12, _16, _18) refers to the dataset (ALS12, MSL16, SPL18) from which the feature was calculated.

Combining all the features from all the systems improved the accuracy in all cases but one (type discrimination using all available features). This improvement, in general, was about 5% compared to using MSL only, except for the classification of tree type. Figure 4 shows the feature rankings for the 12 species-all sensors-all features model that were ordered by *Mean Decrease Gini* and which were produced with the *varImpPlot* function of the Random Forest package in R. The Mean Decrease Gini (unitless) is the mean of a feature’s total decrease in node impurity, weighted by the proportion of samples reaching that node in each individual decision tree in the Random Forest. It is a measure of how important a feature is for classification accuracy across all the trees in the Random Forest. The relative ranking of the features is of interest in these Figures. The suffix following the variable name of each feature refers to the dataset from which the feature was calculated. Slope features figured amongst the most important, as did both green channel-based multi-spectral vegetation indices. The first return intensity dispersion coefficient from standard ALS is the most significant feature for the 12 species classification. Figure 5 and Figure 6 break down in order of importance the features by 3D and intensity, respectively.

In most cases, more parsimonious classification models, i.e., using only the best 25 or 15 features, displayed only a slight decrease of accuracy compared to using all of the pre-selected features. This decrease was very small (≤0.5%, except for SPL) for the type level, and more apparent, while rarely exceeding 2% for the other classification systems. Our results indicate that the more complex sensors (MSL and SPL) did not substantially improve the performance of our models, with the SPL models being the least accurate in all cases.

## 5. Discussion

### 5.1. Factors Influencing Tree Identification Accuracy

The results that are presented here represent the first time that the classification accuracy of automatically delineated ITC was directly compared amongst single ALS, multispectral ALS and SPL systems. The main factors influencing the classification accuracy include system type (ALS, MSL, SPL), the type of feature that is being used (3D, I) and the number of species classes that need to be identified. The richer I feature set that was provided by the three-channel MSL (26, compared to 8 for ALS, and 6 for SPL) resulted in higher classification accuracies across all cases than using 3D features only. This is consistent with results that were found by [6,36] using the same MSL sensor. The best results across all the system types are obtained when combining 3D and I features with the MSL system, which once again featured the highest accuracies of all three system types. The MSL overall feature set (45) was also richer compared to ALS (31) and SPL (23). The NDVI-like features that were provided by MSL consistently emerged as the top 10 most important features that are selected by the Random Forest models (e.g., Figure 4 and Figure 6). Furthermore, larger numbers of features increase the number of features that remain after the correlation filter is applied, which provided more information when training our model.

SPL’s higher point density does not seem to mitigate its limitations when classifying species. As shown in Figure 2 (bottom), the point cloud that was provided by SPL over dense canopy is more akin to the photogrammetric point clouds that are obtained through stereo image matching, with most of the returns being provided by the uppermost part of the canopy and composed of singleton returns. The distribution of returns (first vs. second) is very different from that for linear ALS, with MSL having almost four times the number of pulses with multiple returns than does SPL [46]. A possible explanation for this observation is that data acquired with SPL systems require extensive noise removal for daytime acquisitions [31]. Most methods for noise suppression in SPL are based upon the elimination of isolated points, which potentially removes signal photons. The remaining points are clustered and, therefore, are likely to be redundant. Spurious return filtering is not required for linear ALS systems (except for the occasional very high or low points). Lastly, the positional precision of the SPL sensor (Leica SPL100, Leica Geosystems Ltd. (North America), Lachine, QC, Canada) has been shown to be weaker than that of the MLS sensor (Optech Titan, Teledyne Optech, Toronto, ON, Canada) that was used in this study [46], which may lead to the “blurring” of 3D features.

As was the case for the intensity features that were derived from the linear ALS, the precise interpretation of the intensity values that were provided by SPL was also problematic for reasons similar to those evoked for 3D features. Linear ALS systems detectors produce voltage that is linearly proportional to the number of photons being recorded [30]. There are still many unanswered questions regarding the algorithms that are used to calculate ALS intensity values (they are proprietary to the various instrument manufacturers) and they preclude the comparison of lidar acquisitions that are provided by different sensors and over-flights. SPL detectors yield a binary response to incoming photons, theoretically precluding the calculation of an intensity value for each (single photon) return. It is thus approximated by computing a measure of local point density for each cloud [57]: the detector in the SPL system can register multiple single-photon hits (from the same pulse) in each channel and sum the output to form an analogue value of intensity for each return [46]. This ambiguity exacerbates the existing limitations of using linear ALS intensity values in classification models, as described above.

Comparing our results to those of other studies is difficult, given that most (80%) of the 97 studies that were analysed in a review by [56] classified four or fewer species classes. Furthermore, several species identification papers use a manual or semi-manual process for delineating crowns and combine other datasets, such as optical and hyperspectral imagery, with the lidar data (e.g., [58,59]. Additionally, there are very few species identification studies using MLS or SPL systems at the individual tree crown level. Comparing study accuracies relative to each other is difficult since the number of species, species included, the type of forest biome, and different acquisition parameters can vary so much between studies. The Number of Categories Adapted Index (NOCAI) has been proposed as a means of enhancing comparability between tree species classification studies [60]. It is calculated by dividing the accuracy that is obtained for a given model by the expected accuracy of randomly assigned tree species. The expected accuracy is modelled by 1/*k*, with *k* representing the number of species classes for a given study. Higher values of NOCAI indicate a better performance by the classifier.

The authors of [36] achieved an accuracy of 76% (NOCAI = 7.6) for 10 similar species and 95% (1.9) for type (HW/SW), while [54] obtained a similar accuracy of 77% (7.7) for 10 species in Sweden, and [42] obtained an accuracy of 88% (5.3) on a subset of 6 needle-leaved species, with all of these studies using the same MSL system. The results for the best 12 species classification that were obtained in this study was 58% (7.0). While the accuracies in the aforementioned papers are much higher than the ones that were obtained in this paper for the highest number of species classes, one difference that distinguishes them from our method is the manual delineation of tree crowns rather than automatic delineation that was used in this paper. Furthermore, the aforementioned studies used nearly double the number of training crowns that were used in our study. However, the results for the coarser level groupings, the functional group 75% (3.8), and HW/SW 91% (1.8) are consistent with the studies mentioned above.

The authors of [6] achieved 86% (2.6) accuracy and [60] achieved 89% (2.7) accuracy for three species in Finland, once again using the same MSL sensor. Notwithstanding the difference in forest structure between Finland and Canada, which makes automatic delineation less challenging to perform accurately, our results at the four-genera level with MSL at 79% (3.2) accuracy compare with the results obtained in these two papers, especially as most of our genera classes contained more than one species. Our results differ from those reported in the former papers, in which the authors found that MSL performs better than ALS when more species were classified. More generally, our results are consistent with the survey by [56], who found that across numerous studies, classification accuracy decreases with the number of species classes being considered. In addition, the average NOCAI that was calculated for the best-performing studies compare favourably to those that we obtained in the two-species (1.9 in review average vs. 1.8 in this paper) and four-species (3.4 vs. 3.2) cases. Our Functional Group model with 3.8 performed in a manner similar to the five-species classification review average of 4.0.

The inferior performance of SPL for species identification that was found in this paper is contrary to studies that are based upon ABA, which found that SPL is comparable to MSL [34] and ALS [61] when it comes to calculating forest inventory parameters (e.g., Lorey’s height, basal area, stem volume, aboveground biomass). This difference can be explained by the fact that ABA uses statistical methods that are based upon the height distribution of the lidar returns, rather than the type of return (first vs. second under our method used in our example). The discrepancies in penetration depth between ALS and SPL are not exploited under ABA. Furthermore, a classification problem (species identification) is fundamentally different from modelling structural attributes with regression models, or others.

When combining the features from all three datasets (last column of Table 6), we see that it improved results by about 5% in all cases, except for the type classification (HW/SW). Each system likely provides a specific type of information content that is not redundant or repeated between systems, thereby increasing classification accuracy. An additional, or alternative, explanation is that having thus created a 6 year time series of data, perhaps inter-species differences in feature evolution (e.g., specific growth patterns) are captured as well. Even though multiple acquisitions on the same area by these three different systems may not be economically recommended, the temporal aspect may have made multiple acquisitions by standard ALS systems more useful as additional data for our models.

When examining the relative importance of features for the 12 species classification using the combined dataset (Figure 4), slope-based features were the major single contributor. However, intensity features composed most of the top 10. It should be noted that the two MSL green channel vegetation indices (I_G_IR1 and _IR2) appeared in the top 10 features. SPL contributed the least number of features, i.e., two. When looking at 3D features only (Figure 5), slope-based features contributed significantly, as did convex hull features from the ALS and MSL datasets. Given that the SPL data are mainly composed of first returns near the top of the canopy, a convex hull value was not computed for many crowns, reducing the value of the THREED_CH_18 feature. SPL once again contributed the least number of features to the model: two. Figure 6 shows the intensity features in isolation. Three MSL-based spectral indices were amongst the top 10 features of the model; features that were based upon the ratio of median intensity values between first and second returns contributed to the model as well. Finally, the most significant feature in the combined model and the intensity-only model was the coefficient of dispersion of first return intensity values (I_DI_1st_sd_12) from the ALS dataset.

### 5.2. Implications for Forest Inventory

Generally, the most requested output from the remote sensing acquisitions of forests consists in the species-specific size distributions of their individual trees [6]. The results that are presented in Table 7 for the 12 species classification (58% using 97 features from the combined datasets) fall short (with 70% being a reasonable threshold, in our opinion) of being sufficiently accurate for operational use. There was also a clear difference amongst most hardwood and softwood accuracies, with the accuracy of most softwood species being much higher (≥60%, except for LA) compared with hardwood species (≤40%). This illustrates the continuing challenge of accurate tree crown delineation and identification in dense, mixed hardwood forests.

Higher accuracies have been achieved through the fusion of hyperspectral imagery and ALS. For example, an accuracy of 88% was obtained for eight savannah tree species [62]. The delineation of savannah trees is greatly facilitated when compared with dense natural forests. However, there are geometric and radiometric registration challenges when two different sensors are used, given that data are usually acquired at different times and from differing viewing geometries between lidar and optical systems [63,64]. Evidently, automated delineation is required for operational use. Errors of commission and omission arising from delineation, and the difficulties of identifying training crowns in the field, or label noise (see next section), are all factors that affect classification accuracy when using machine-learning classifiers such as Random Forest.

Linear ALS systems are now widely used to provide the structural information that is used to construct enhanced forest inventories, specifically with the ABA [8]. Cost is an important factor to consider, due to the large areas that need to be covered operationally. The ground that is sampled by an ALS sensor at any given time is a function of flight altitude, speed, and maximum scan angle. For ALS systems, there is a direct relationship between point density and cost. If a sensor, such as SPL, can cover more km^2^ h^−1^ at the same theoretical point density, then there is a clear cost advantage in using SPL, at least in the case of the ABA [34,61]. As demonstrated by our results, there are apparent differences in the point cloud that was produced by linear ALS and SPL systems, resulting in lower accuracies across the board for SPL acquisitions. The structure of the returns (far fewer second returns) arising from the lower penetration of the canopy achieved by the SPL system is different when compared to linear systems [65]. When combined with the fact that features using the ratio of first vs. second returns are frequently retained in our models, this results in the lower accuracies that were recorded for the SPL system.

### 5.3. Limitations and Research Avenues

Our study revealed some limitations when we tried to apply machine-learning methodologies to a natural environment and on a large scale. The first limitation concerns the sparseness of our training data. Machine-learning classifiers, such as Random Forest, show a corresponding increase in accuracy when the sample size is increased [66,67,68]. At the 12 species classification level, some species consist of 35 exemplars, which is a very small number when compared to typical machine-learning image classification problems, where each class features tens of thousands (millions in the case of deep learning) of exemplars for each class [69]. Without mitigation measures such as feature selection (this is especially true when using balanced Random Forest models, as in this paper), the paucity of our training data would also lead to issues that are related to the aforementioned curse of dimensionality, since the ratio of training crowns to calculated features would be far too low.

The noisiness, or occasional mislabelling, of our training data is another limitation of this study. The software that was used to delineate the crowns attempts to assign a precise delineation to detected tree-tops to produce the crown polygon layer depicting theoretical crowns. This layer is then used (as discussed in Section 3.3.1) to identify training crowns and to assign a species to them. Several sources of systematic error are then introduced into the model: GPS drift and difficulties in spatial orientation, which originates from relating the crowns that are generated by SEGMA to the canopy that is observed by looking upward, mean that some training species are obviously mislabelled, or suffer from label noise [70], such as two entwined crowns growing together. An additional source of training crown impurity is delineation error, especially at the edges of the crowns. If delineation is not exact, then there can be different species that are included in the training crown around its edges. Although Random Forest is shown to exhibit robustness to label noise [71], higher levels of label noise exert a subsequent negative effect upon classification accuracy [72].

As mentioned in the previous section, there are possible temporal decorrelation issues that are related to the training data acquired in 2012, while SPL was acquired in 2018. The strong results that were obtained from the MSL 2016 acquisition mitigate this possibility. The differences between SPL and linear ALS data for species classification at the individual crown level need to be investigated further, together with accuracies that must be improved across the board, to become operational at large scales. An encouraging observation from this study, however, shows that training crowns that are acquired in one year can be used in subsequent acquisitions, even when accounting for the usual intensity standardisation problem between different lidar sensors, and even with the same instrument. This shows the potential for building a library of training crowns that would be usable across different datasets when accuracy levels become high enough to be operational.

To bring forward actionable species information for enhanced forest inventories at the individual crown level, future research should concentrate on improving the delineation process. Improvements in the accuracy of the delineation process should translate directly to enhanced accuracy of tree species identifications at all levels of fineness. We can also ask ourselves whether we need to delineate the entire crown exactly; perhaps a circular (or other shape) buffer around radius of a given distance from the local maximum could provide features that suffer less from the label noise effects that are caused by uncertain crown edges than are experienced currently. The temporal effect species signal that may exist for features calculated across multiple data acquisitions should also be further investigated. This does not require three types of sensors to capture this signal per se, but it would be interesting to observe whether just two ALS over-flights that are separated by a few years exhibit the same behaviour as found in this study. To reach its maximum potential usefulness, more must be known about lidar intensity (across all systems) to be able to standardise the values across acquisitions. This would surely increase the already significant classification power of intensity features for species identification.

## 6. Conclusions

This paper compared the performance in tree species identification achieved by three different lidar systems, including multispectral and single-photon instruments, at the individual tree crown level, using the same training crowns and methodology across the three datasets.

MSL provided the greatest species identification accuracy across all the groupings, while SPL displayed the lowest. In the case of the combined dataset, MSL provided more intensity features, while ALS and SPL provided mostly 3D features. When the results were broken down by feature type (3D vs. I), we found that geometric features performed better than intensity features for the monospectral linear and single-photon instruments. As expected, the enhanced intensity features of linear multispectral lidar performed better than the geometric features, even with the enhanced point density that had been acquired by the three laser beams in that particular instrument. In all cases, the combined geometric and intensity features performed the best. Single-photon lidar intensity features performed the poorest across all datasets. Interpreting this result is made difficult by the fact that the derivation and meaning of the SPL intensity measurements is still not well described in published research.

In dense mixed forests such as PRF, hardwoods remain a classification challenge at the 12 species classification level, while softwoods are classified more accurately. Hardwoods are more challenging to delineate accurately and are more prone to identification error when selecting training crowns in the field. The low number of exemplars in certain species classes lowered the effectiveness of the Random Forest classifier, since all classes would have their training data limited by the class with the lowest count.

The fact that training crown polygons were segmented and field-sampled in one year (2012) and used in subsequent lidar over-flights (2016 and 2018) is encouraging, as it means that fieldwork does not have to be duplicated to use a more recent acquisition. A novel combination of all three dataset features in a single classification model, which improved accuracy by an additional 5% in most cases, was performed as well. The success of this combination suggests that multi-temporal species differences between features arising from multiple lidar acquisitions would not necessarily have to originate from three different types of sensors, as were used in this study, but these differences in features could contribute to accuracy improvement, which merits further investigation.

## Figures and Tables

**Table 1:** Acquisition parameters and information for lidar datasets.

Parameter	ALS12	MSL16	SPL18
Acquisition date	17–20 August 2012	20 July 2016	1–2 July 2018
Sensor	Riegl 680i	Optech Titan	Leica SPL100
Laser wavelength (nm)	1550	532/1064/1550	532
Laser beam divergence (mrad)	0.5	0.7/0.7/0.35	0.08
Avg. flying altitude (m AGL)	750	600	3760
Avg. flying speed (kts)	<100	<140	<180
Pulse repetition frequency (kHz)	150	375 (3 channels)	60
Frequency (Hz)	76.67	40	23
Scan angle (degrees)	±20	±35	±15
Field-of-View (degrees)	40	30	30
Aggregate point density (points/m^2^)	5.8	4.8/12.4/11.9 ~30 (combined)	28.6

**Table 2:** Description of 3D-based features for each crown (prefix = 3D_).

Symbol	Description	Return Types	Statistics
DI	Dispersion (coefficient of variation of return heights)	all, 1st	cv
SLOPE	Slope of the lines connecting the highest return to each of the other returns	all, 1st	mn, sd, cv, p25, p50, p75
RB	Ratio of the number of returns in different height bins (% of height) over total number of returns	all	Counts: 60_80, 80_90, 90_100, 95_100
CH	Ratio of the convex hull volume over maximum height cubed	all	N/A
RM	Ratio between different statistics and different types of return	all, 1st, 2nd	mn, p50

Abbreviations: all = all returns, 1st = first returns, cv = coefficient of variation, mn = mean, sd= standard deviation, p = percentile.

**Table 3:** Description of intensity-based features for each crown (prefix = I_).

Symbol	Description	Return Types	Statistics
DI	Dispersion (coefficient of variation of intensity)	1st	sd, cv
PE	Intensity values at given height percentiles	1st	p5, p10, p25, p50, p75, p90, p95
MI	Mean intensity of returns between interval of percentiles	1st	mn: all, p5_95, p10_90,
RM	Ratio between different statistics	all, 1st, 2nd	mn, p50
G_IR1 (MSL)	Type 1 Green Normalised Difference Vegetation Index (532 nm and 1064)	1st	mn, p50, p75
G_IR2 (MSL)	Type 2 Green Normalised Difference Vegetation Index (532 nm and 1550 nm)	1st	mn, p50, p75
NDIR (MSL)	IR Normalised Difference Vegetation Index (1064 nm and 1550 nm)	1st	mn, p50, p75
	Simple ratios of 3 MSL wavelengths	1st	mn, p50, p75

Abbreviations: all = all returns, 1st = first returns, 2nd = second returns, cv = coefficient of variation, mn = mean, sd= standard deviation, p = percentile.

**Table 4:** Number of training crowns per species ALS12 (*n* = 1413).

Species	*n*
Black ash	45
White ash	40
Basswood	56
Beech	70
Balsam fir	78
Paper birch	53
Yellow birch	40
Eastern white cedar	44
Eastern larch	46
Sugar maple	137
Red maple	54
Red oak	72
Jack pine	89
Bigtooth aspen	100
Red pine	109
Trembling aspen	48
White pine	159
Black spruce	65
White spruce	108

**Table 5:** Training crown groupings.

HW/SW	ALS12	MSL16	SPL18
Hardwood	683	614	596
Softwood	673	566	546
**Four Genera**			
*Acer* (maple)	185	175	155
*Pinus* (pine)	345	280	302
*Populus* (poplar)	135	102	139
*Picea* (spruce)	171	154	130
**Functional Group (Fct. Gr.)**			
Hardwood	308	297	262
Intolerant hardwood	375	317	334
Other softwood	157	132	114
Pine	345	280	302
Spruce	171	154	130
**12 Species**			
Ash (Black/White) (AS)	81	70	66
Basswood (BA)	56	53	48
American Beech (BE)	67	69	59
Birch (Paper/Yellow) (BI)	89	80	77
Eastern White Cedar (CE)	44	43	36
Balsam Fir (BF)	69	53	43
Eastern Larch (LA)	44	36	35
Maple (Red/Sugar) (MA)	185	175	155
Red Oak (OK)	70	65	52
Pine (Red/White) (PI)	345	280	302
Trembling Aspen (PO)	135	102	139
Spruce (Black/White) (SP)	171	254	130

**Table 6:** Random Forest classification accuracy in % (20 runs) broken down by 3D and intensity (I) features, pooled by system (All), and pooled across all systems and features (ALL ALS, All).

		ALS12			MSL16			SPL18		ALL ALS
	3D	I	All	3D	I	All	3D	I	All	All
**Type (HW/SW)**										
All features	84.0	76.2	86.4	82.9	85.2	90.4	80.1	59.1	82.9	90.3
25 features	N/A	N/A	86.1	N/A	85.2	90.4	N/A	N/A	N/A	91.1
15 features	84.1	N/A	86.3	82.9	85.0	89.9	80.1	N/A	82.7	91.0
**4 genera**										
All features	65.4	64.7	75.1	67.8	71.7	78.6	64.5	50.1	68.3	83.4
25 features	65.5	N/A	74.3	N/A	71.8	78.1	N/A	N/A	N/A	83.5
15 features	63.6	N/A	74.2	66.4	71.8	76.8	63.5	N/A	68.1	81.4
**Functional Group**										
All features	55.7	52.5	68.9	54.3	64.1	69.6	50.4	43.3	63.6	75.0
25 features	N/A	N/A	67.4	N/A	64.3	69.2	N/A	N/A	N/A	73.0
15 features	55.3	N/A	66.2	54.1	64.1	68.6	50.0	N/A	64.4	72.5
**12 species**										
All features	38.9	33.2	50.7	36.8	48.2	53.2	37.8	25.8	44.5	58.0
25 features	N/A	N/A	51.3	N/A	48.5	53.5	N/A	N/A	N/A	57.0
15 features	38.3	N/A	48.7	37.3	47.5	51.5	38.4	N/A	44.8	54.2

**Table 7:** Confusion matrix for 12 species classification using best model (all sensors, all features). Definitions of the two-letter acronyms for tree species are given in Table 5, softwood species in grey.

	**AS**	**BA**	**BE**	**BI**	**CE**	**BF**	**LA**	**MA**	**OK**	**PI**	**PO**	**SP**	**OOB Accuracy %**
**AS**	**22**	4	1	5	0	0	1	5	9	4	4	1	**39**
**BA**	10	**13**	3	2	0	0	1	3	4	5	4	1	**28**
**BE**	1	0	**36**	2	0	0	0	14	0	0	2	1	**64**
**BI**	7	1	4	**21**	3	0	0	6	9	5	10	1	**31**
**CE**	4	1	0	1	**26**	0	1	0	0	0	0	3	**72**
**BF**	0	1	1	0	2	**22**	1	0	2	0	0	5	**65**
**LA**	0	0	0	0	4	0	**10**	0	0	4	0	11	**35**
**MA**	4	5	28	5	1	1	0	**81**	2	7	10	2	**56**
**OK**	5	2	0	3	0	0	0	2	**31**	1	2	1	**66**
**PI**	3	6	0	1	13	4	10	1	2	**185**	16	10	**74**
**PO**	11	5	4	10	4	4	1	2	3	6	**42**	4	**44**
**SP**	0	3	0	0	5	7	3	1	0	7	3	**88**	**75**

## Data Availability

Not applicable.
